# Development of Electrodeposited Wire Mesh Grinding Wheel for Cutoff and Grooving Carbon Fiber Reinforced Plastic

**DOI:** 10.3390/ma16155247

**Published:** 2023-07-26

**Authors:** Mamoru Nomura, Shuji Kurashige, Yukio Ito, Yoshiya Fukuhara, Hiroyuki Sasahara

**Affiliations:** 1 Department of Mechanical Systems Engineering, Tokyo University of Agriculture and Technology, Tokyo 184-8588, Japan; mamoru.nomura@ibarakiseito.com (M.N.); kurashu004427@gmail.com (S.K.); 2 IBARAKI GRINDING WHEEL Co., Ltd., Ibaraki 300-2521, Japan; jms007@qc.commufa.jp; 3 Mitsubishi Heavy Industry, Hyogo 676-0008, Japan; yoshiya.fukuhara.25@mhi.com

**Keywords:** CFRP, wire mesh grinding wheel, clogging, temperature, surface roughness, diamond abrasive grain

## Abstract

Carbon fiber reinforced plastic (CFRP) is used in various industries because of its high specific strength, but it is well known as a difficult material to cut. In this study, we developed a disc-shaped electrodeposited diamond wire mesh grinding wheel as a new method for cutoff and grooving with a large aspect ratio for CFRP. We confirmed that this tool could be used for machining at a feed rate of 1000 mm/min, equivalent to that of an abrasive waterjet. This tool discharges generated chips through the spaces in the wire mesh, preventing clogging and thereby enabling the suppression of machining temperature. No burrs or delamination were observed on the surface machined with the wire mesh grinding wheel, and the surface roughness was *Ra* = 2.76 µm. However, the groove width was larger than the wheel thickness due to the runout of the wheel. Additionally, the moderate elasticity and durability of the tool suggest that it might extend tool life by avoiding the crushing of abrasive grains.

## 1. Introduction

Carbon fiber reinforced plastic (CFRP) is widely used in various industries due to its high specific strength and high specific modulus. Demand for CFRP is increasing, especially in the aerospace industry [1,2]. However, CFRP is known as a difficult material to cut, and aircraft components require a lot of cutoff and grooving operations. Cutting with an end mill, which is a common machining method, has the following problems: machining efficiency cannot be increased to prevent delamination of CFRP fibers [3,4,5,6,7], and tool life is short due to high wear rates [8,9,10,11,12]. Abrasive waterjet processing has also been used [13,14,15], but it has the problem of high initial investment, maintenance, and running costs [16]. In addition, most of these previous studies focused on the cutting or edge-trimming process; there have been few studies on grooving with a large aspect ratio. In this study, we investigate a grooving method with long tool life and low initial investment.

Grinding is widely regarded as a promising machining process for achieving high-quality results when working with CFRP. Hu et al. [17,18] dry-ground CFRP using a resinoid grinding wheel with WA abrasive grains and showed the relationship between surface roughness and the angle of the fibers relative to the grinding direction. The most pronounced surface roughness was observed when the fiber orientation resulted in an obtuse angle between the grain and the cutting direction. This can be attributed to the cutting of fibers as they are lifted during the process.

Diamond abrasives are generally used for grinding CFRP due to their high thermal conductivity and hardness. Gao et al. [19] constructed a mechanical model of diamond single-particle grinding and verified it experimentally, considering the differences in the coefficient of friction between abrasive grains and CFRP under different lubrication conditions. The results showed that minimum quantity lubrication (MQL) grinding can reduce the grinding force compared to dry grinding. Both Wang and Ning et al. [20,21] showed that when rotary ultrasonic machining (RUM) is applied to a metal-bonded diamond grinding tool, the frictional force decreases, and the cutting force and torque decrease because of the shorter contact time between the workpiece and the tool with increasing ultrasonic power. Yuan et al. [22] showed that it is possible to cut CFRP at high cutting speeds (76 m/s, 25,000 rpm) using a diamond-abrasive ultrathin dicing blade. However, the serious vibration of the spindle in the horizontal direction limited the rotational speed to increase further. In addition, the depth of the cut they performed was 0.6 mm, which was rather small for high-efficiency machining. In contrast, our proposed tool can cut and groove with a cut depth of more than 7 mm.

While grinding is expected to produce high-quality workpieces, problems related to clogging are difficult to cut. Clogging problems should be considered because they increase grinding force and generate abnormal temperature increases. Soo et al. [23] showed that tool wear, grinding resistance, and machined surface roughness were lower when diamond abrasive was used in end-face grinding of CFRP than when CBN was used. They also observed that excessive clogging shortened tool life.

Suzuki et al. [24] developed a tool with diamonds electrodeposited on a cylindrically rolled wire mesh as a method to prevent clogging. The tool was applied to the drilling of CFRP, revealing that it could achieve a high-efficiency equivalent to a twist drill. In this study, we took advantage of this feature to develop a tool with diamond abrasive grains electrodeposited on a disc-shaped wire mesh. This tool is characterized by its ability to machine grooves with large aspect ratios and by its long life. In this paper, we show that clogging can be avoided and that low stiffness is effective in extending the life of grinding wheels.

## 2. Wire Mesh Grinding Wheel Features

### 2.1. Appearance and Advantages of Grinding Wheels

**Figure 1:**
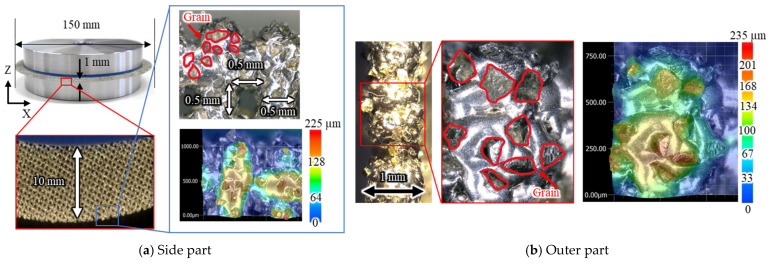
Diamond electrodeposited wire mesh grinding wheel.

**Figure 2:**
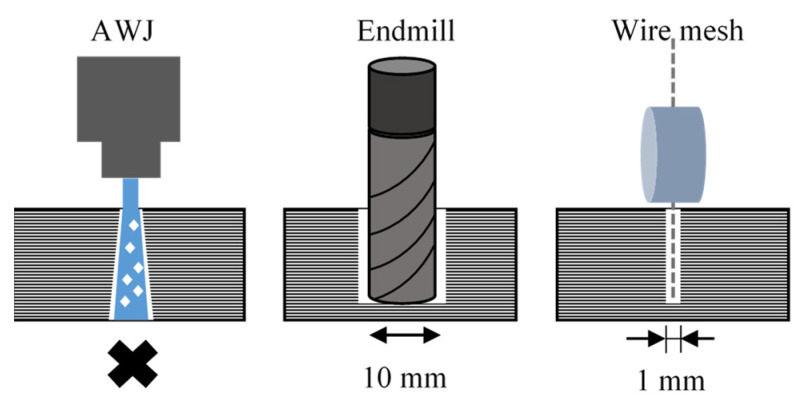
Comparison of grooving methods.

Figure 1 and Figure 2 show the side view of the proposed tool and its outer circumference, respectively. Diamond abrasive grains were electrodeposited around the wire mesh corresponding to the base metal. The diameter was 150 mm, the protrusion from the base disk was 10 mm, and the thickness was 1 mm. The wire is austenitic stainless steel (SS304), 0.5 mm in diameter, with a space of 0.5 × 0.5 mm. The grain size was #100 (average grain size 149 µm). The mesh size depends almost entirely on the wire diameter. In addition, a space must remain after the abrasive grains are electrodeposited. After considering these factors comprehensively, a space size of 0.5 mm × 0.5 mm was selected.

In addition, the wire mesh grinding wheel had peripheral runout, which is difficult to remove. One of the reasons for this is the low roundness of the wire mesh when it is cut into disk shapes. Nevertheless, as will be described later, cutting is possible without any problem, even if there is a large runout.

**Figure 3:**
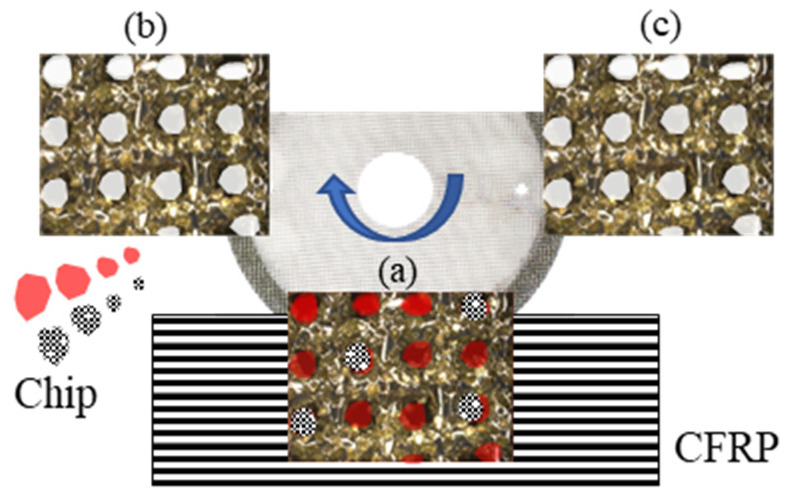
Schematic diagram of chip discharge process. (**a**) Chips are temporarily held in the wire mesh space, (**b**) Chips are discharged, (**c**) Free of clogging before engaging.

Figure 2 shows the difference in groove width when machining CFRP using an abrasive waterjet (AWJ), an end mill, and a wire mesh grinding wheel. AWJ is not suitable for grooving because it only allows machining through penetration. A wire mesh grinding wheel can be used to machine thin and deep grooves with larger aspect ratios than end mills. Although such grooving is possible with general electroplated cutters, the difficulty in removing chips results in frequent tool changes due to clogging. In the case of a wire mesh grinding wheel, the chip discharge effect shown in Figure 3 can prevent clogging. In the part of the grinding wheel involved in machining, chips are temporarily held in the wire mesh space, as shown in Figure 3a. In the part of the grinding wheel not involved in machining, chips are discharged by centrifugal force due to rotation, as shown in Figure 3b. The pre-processed part of the grinding wheel remains free of clogging, as shown in Figure 3c.

### 2.2. Stiffness of Grinding Wheel

**Figure 4:**
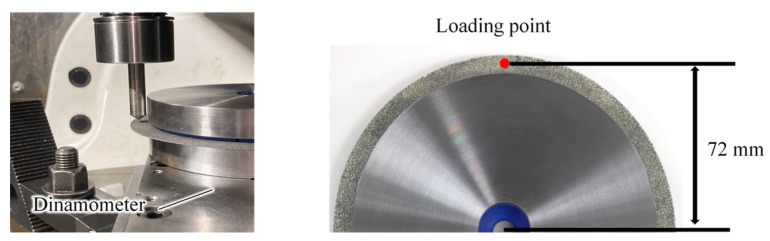
Experimental setup.

**Figure 5:**
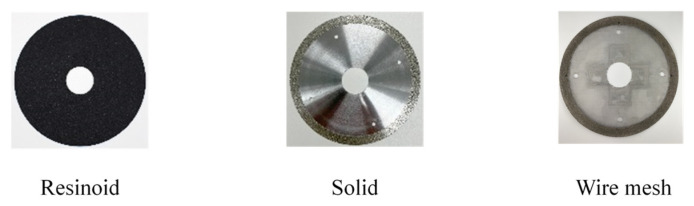
Grinding wheels.

The base metal of the proposed tool was wire mesh, which is less rigid than a typical solid base metal, even considering that the electrodeposition layer strengthens the wires by bonding them to each other. We first investigated the stiffness of this wire mesh electrodeposited grinding wheel. Figure 4 shows the method of testing for out-of-plane bending stiffness. A wheel was fixed on a dynamometer (KISTLER 9257B), a pin was pressed down 72 mm from the center against a wheel with a diameter of 150 mm (radius 75 mm), and displacement and load were measured. In addition to the electrodeposited diamond mesh wheel (hereafter referred to as the wire mesh wheel), the bending stiffnesses of two other wheels were measured for comparison: a resinoid wheel (grain: GC, grain size: #100, bond: N) and an electrodeposited diamond solid wheel (base metal: SUS304, grain: diamond, grain size: #100, hereafter referred to as the solid wheel). Figure 5 shows these three types of grinding wheels.

**Figure 6:**
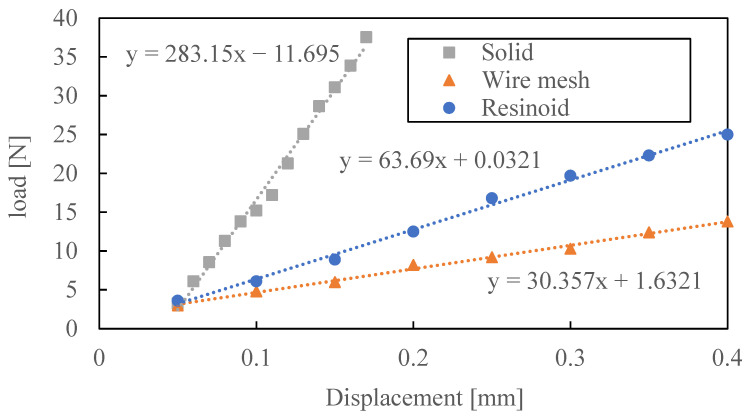
Relationship between bending load and displacement of each grinding wheel.

Figure 6 shows the relationship between load and displacement for each grinding wheel. The load varies linearly with displacement for all grinding wheels. As the slope of the graph is the stiffness of each wheel, the stiffness values of the resinoid wheel, the solid wheel, and the wire mesh wheel were 63.7 N/mm, 283 N/mm, and 30.4 N/mm, respectively. The stiffness of the wire mesh wheel was about 1/2 that of the resinoid wheel and 1/10 that of the solid wheel.

### 2.3. In-Plane Stiffness of Thin Grinding Wheel

**Figure 7:**
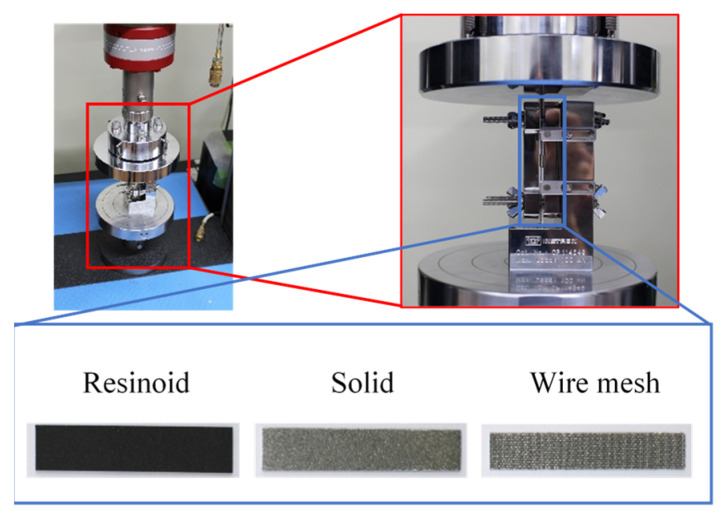
Experimental setup.

The in-plane stiffnesses of thin grinding wheels were also tested as follows. Figure 7 shows a photograph of the experimental setup. In-plane compression tests were conducted using a universal material testing machine in accordance with JIS standard JISK7018. Strain and stress were measured by fixing the bottom and side of each of the three grinding wheel strips shown in Figure 5 and pressing them into the test specimens from above.

**Figure 8:**
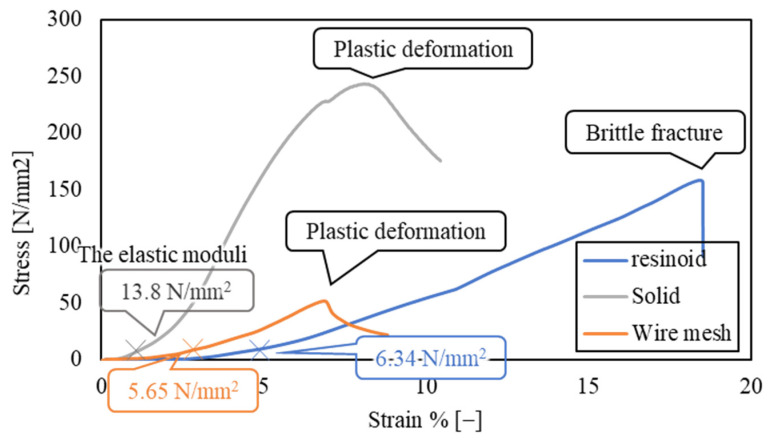
Relationship between compressive stress and strain in thin grinding wheel plane.

Figure 8 shows the relationship between stress and strain for each wheel. After a relatively large elastic deformation of the resinoid wheel, it experienced a brittle fracture, and the stress dropped rapidly at that instant. On the other hand, the solid and wire mesh grinding wheels deformed elastically and then plastically, and after reaching a certain stress, the stress decreased smoothly. The elastic moduli of the wheels against the in-plane load were 6.34 N/mm^2^, 13.8 N/mm^2^, and 5.65 N/mm^2^ for the resinoid, solid, and wire mesh wheels, respectively. The stiffness of the wire mesh electroplated wheel was equivalent to that of the resinoid wheel and less rigid than that of the solid wheel. While low stiffness is a disadvantage in reproducing motion trajectory, it does contribute to extending tool life, as described below. Although not discussed in this paper, it is possible to control the stiffness of the wire mesh by changing the thickness and density of the mesh wires by making the wire mesh multilayered or by using some other method. We think that these issues should be considered in the future.

## 3. Experimental Setup and Method for Cutoff and Grooving

### 3.1. Workpiece

Table 1 shows the mechanical properties of the CFRP used as the workpiece. The CFRP plate was laminated with unidirectional prepreg (QU135-197A manufactured by Toho Tenax, Tokyo, Japan) and hardened to a thickness of 7.6 mm. The specimens were quasi-isotropic, with stacking configurations of 0°, −45°, 45°, and 90°.

### 3.2. Machining Characteristics during Cutting and Grooving

**Figure 9:**
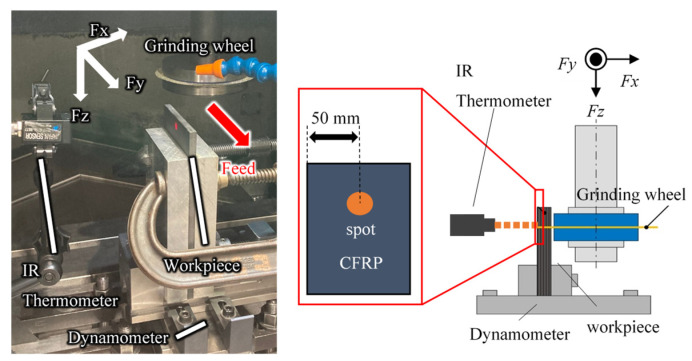
Experimental setup.

Figure 9 shows the experimental setup. A vise was attached to a piezoelectric dynamometer (Kistler 9257B, Winterthur, Switzerland) set on a table of the machining center, and the CFRP plate was fixed vertically. This dynamometer has a stiffness and natural frequency high enough to measure the absolute value of the grinding force and its variation in this experiment. The sampling period in this experiment was 1 ms. Table 2 shows experimental condition A. Comparative experiments were conducted with the three grinding wheels shown in Figure 5 at a cutting speed of 1884 m/min, a feed rate of 100 mm/min, a down-cut wheel rotation direction, and a grinding fluid supply. Coolant was used here because the primary concern was to avoid the disadvantages of high temperatures above the glass transition point. A dynamometer was used to measure the grinding force, and an infrared radiation thermometer was used to measure the temperature at the backside of the machining point. The IR thermometer outputs the average temperature within an 8 mm diameter LED sight. This was supplemented by a thermographic camera to monitor and analyze temperature conditions during the experiment. In addition, the machined surface of the workpiece and the surface of the grinding wheel after machining were observed using an optical microscope.

The use of coolant in cutting CFRP causes cutting oil to permeate the CFRP interlayer and the interface between the fibers and matrix resin, resulting in problems such as reduced strength and dimensional defects. To avoid these problems, dry machining is recommended. For this reason, temperature comparisons were also conducted for dry processing. Table 2 shows experimental condition B. The feed rate was set at 1000 mm/min, the same level as that of the abrasive waterjet, to compare the two grinding wheels shown in Figure 5, the solid grinding wheel and the wire mesh grinding wheel. The processing temperature during the cutting process was measured with a thermographic camera. A resinoid grinding wheel was excluded from the experiment because it is quickly damaged in dry machining.

### 3.3. Tool Life Test with Continuous Grooving

**Figure 10:**
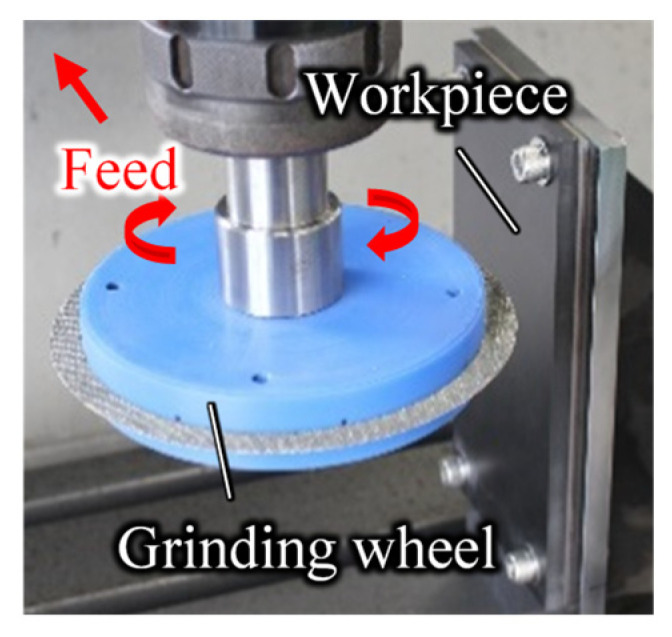
Experimental setup for grooving.

Figure 10 shows the experimental setup. CFRP was fixed with bolts to a jig set on a table at the machining center. The experimental conditions followed Table 2, with a cutting speed of 2120 m/min and a feed rate of 300 mm/min. In the tool life test, the feed rate was set higher than in the force/temperature measurement, aiming to accelerate the wear rate by increasing the feed rate to the point where the grinding wheel is not damaged. Grooves with a depth of 7 mm were machined 60 times on work material 7.6 mm thick, the outer diameter of the wheel was measured, and the surface of the wheel was observed. Because the length of the workpiece was 100 mm, the machining distance for one grooving operation was 100 mm. The three grinding wheels shown in Figure 5 were used for comparison. These wheels were in unused condition at the start of the experiment.

## 4. Results and Discussion

### 4.1. Machining Characteristics during Cutting and Grooving

#### 4.1.1. Observation of Grinding Wheel Surface

**Figure 11:**
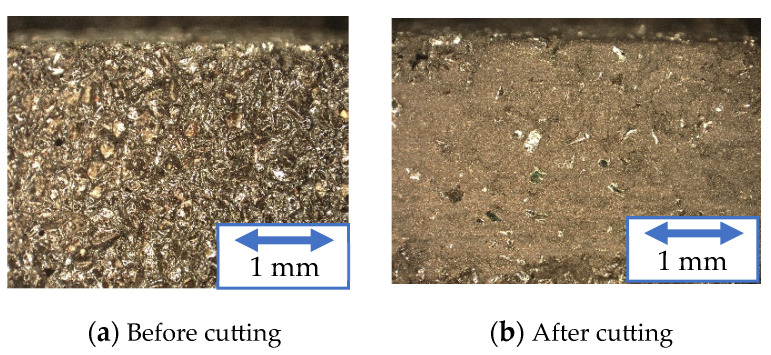
Grinding wheel surface (Resinoid).

**Figure 12:**
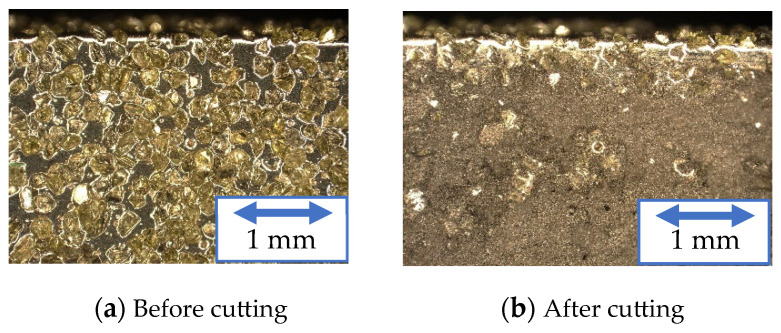
Grinding wheel surface (Solid).

**Figure 13:**
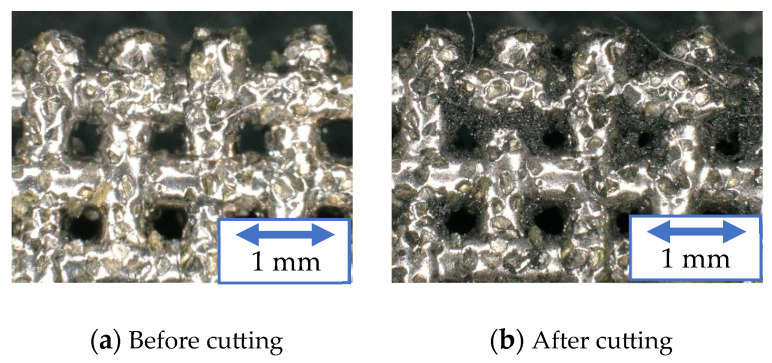
Grinding wheel surface (Wire mesh).

Figure 11, Figure 12 and Figure 13 show the surfaces of the resinoid, solid, and wire mesh grinding wheels, respectively, before and after machining under experimental condition A. The figure on the right shows the grinding wheel after machining 300 mm. Chips stuck to the resinoid grinding wheel and covered the abrasive grains, resulting in clogging. In the solid grinding wheel, clogging was observed on the sides, but the tip was not covered with chips. The wire mesh grinding wheel, on the other hand, had only a few chips stuck to both the sides and the tip. These chips could be easily removed by blowing air, indicating that there was no clogging. This is because the chips were discharged through the spaces in the wire mesh. As for the solid grinding wheel, the chips could be removed by ultrasonic cleaning, but continuous machining could not be expected. On the other hand, continuous long-distance processing can be expected from a wire mesh grinding wheel if it is treated with air.

#### 4.1.2. Grinding Force

**Figure 14:**
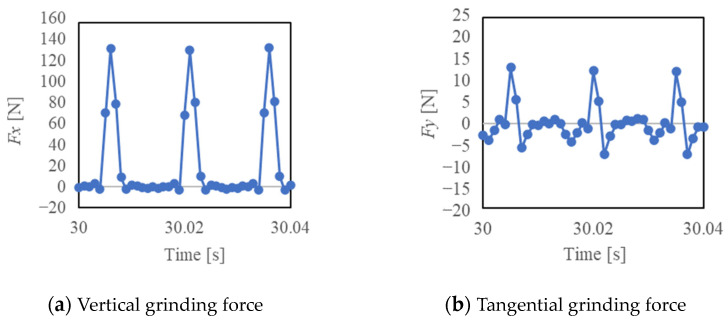
Grinding force (Resinoid, Time: 31.0~31.04 [s]).

**Figure 15:**
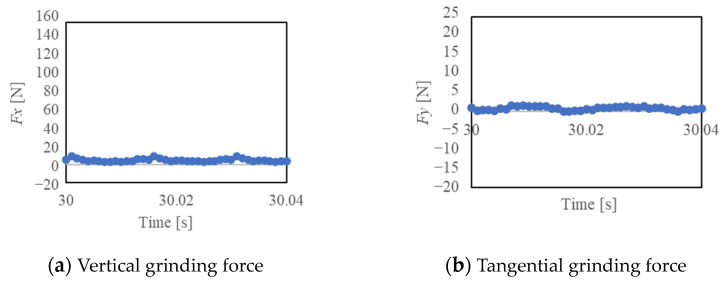
Grinding force (Solid, Time: 31.0~31.04 [s]).

**Figure 16:**
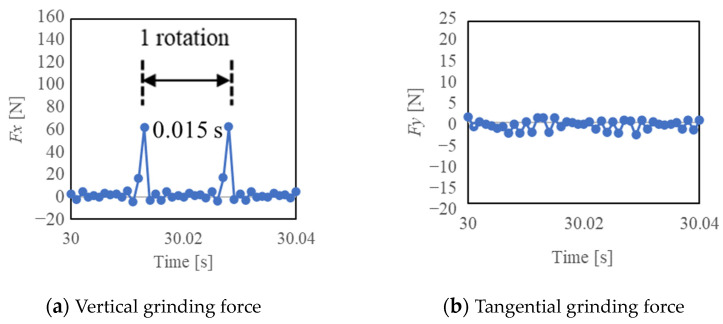
Grinding force (Wire mesh, Time: 31.0~31.04 [s]).

Figure 14, Figure 15 and Figure 16 show the vertical grinding force *Fx* and the tangential grinding force *Fy* for machining with a resinoid, solid, and wire mesh grinding wheel, respectively, under experimental condition A. Grinding forces for several spindle rotations are shown for comparison of the maximum value of the force (Time: 31.0~31.04 s). In the resinoid, the number of abrasive grains appearing on the wheel surface decreased due to clogging, causing the wheel to be pressed against the workpiece, which in turn resulted in a higher grinding force. Comparing the solid and wire mesh grinding wheels, whose tips were not clogged, the wire mesh grinding wheel had a higher grinding force. This is because the wire mesh grinding wheel has peripheral runout, and a large force is applied instantaneously because only a small portion of one revolution is involved in grinding. The sampling period for this experiment was 1 ms, and the spindle speed was 4000 rpm (0.015 s per revolution), so the grinding force was plotted 15 times per revolution. In the waveforms measured with the wire mesh grinding wheel, the force was measured once per revolution. However, this is not a problem because cutting is still possible in this state. On the other hand, the low dimensional accuracy in the *z*-axis direction in Figure 1 is a problem in groove machining, as discussed in Section 4.1. When grooving with a resinoid or solid grinding wheel, the groove width is about 1.1 mm, whereas a wire mesh grinding wheel produces a groove about 1.6 mm wide. Thus, care must be taken when machining.

#### 4.1.3. Temperature behind the Grinding Point and Observation of the Machined Surface

**Figure 17:**
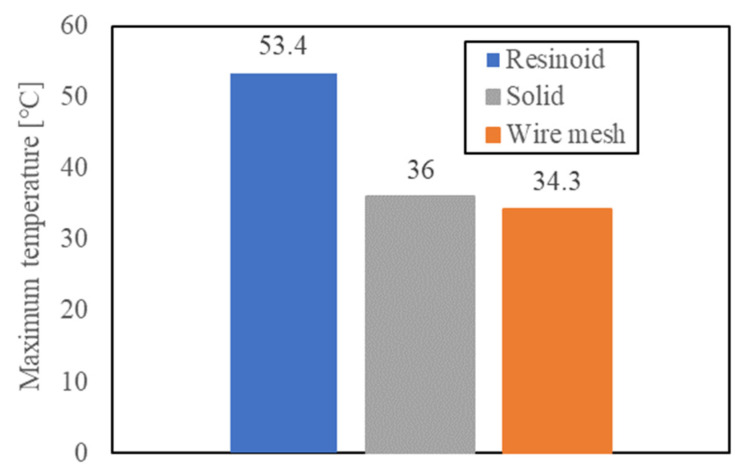
Maximum temperature behind the processing point.

The temperature behind the machining point was measured using an IR thermometer when a groove 7 mm wide was machined in 7.6 mm CFRP. A cylindrical cover was used to prevent grinding fluid from entering the optical path of the IR sensor during the measurement, and it was confirmed that the spot position was not wet after the measurement. Figure 17 shows the maximum temperature measured under experimental condition A. The temperature at the machining point is estimated to be higher because the average temperature in a spot with a diameter of 8 mm is shown for a wheel thickness of 1 mm, and the spot is placed on the backside of the machining point. The maximum temperatures of the resinoid, solid, and wire mesh grinding wheels were 53.2 °C, 36.0 °C, and 34.3 °C, respectively, under condition A. The processing temperatures of the wire mesh and resinoid grinding wheels differed by approximately 20 °C. This is because the abrasive grains in the wire mesh grinding wheel always did their work due to the suppression of clogging during machining, whereas the abrasive grains in the resinoid wheel tended to become covered with chips due to clogging during the machining process, thus reducing the effectiveness of the abrasive grains and increasing friction. Consequently, a larger temperature rise was observed in resinoid wheels compared to wire mesh grinding wheels. The processing temperature of the solid grinding wheel was higher than that of the wire mesh grinding wheel. This is presumably because it is difficult for the grinding fluid to reach the inside of the groove with a solid grinding wheel, whereas a wire mesh wheel can temporarily hold the fluid in its mesh and supply it to the machining point.

However, the temperature difference between the solid and wire mesh grinding wheels was not significant. As described in Section 3.2, the temperature behind the machining point was measured instead of the machining point, and the measurement area of the infrared thermometer was Φ8 mm, which was wider than the grinding wheel width. These are the causes of lower temperature output than the actual temperature. Therefore, a new comparison was made in dry machining with a feed rate of 1000 mm/min (experimental condition B).

**Figure 18:**
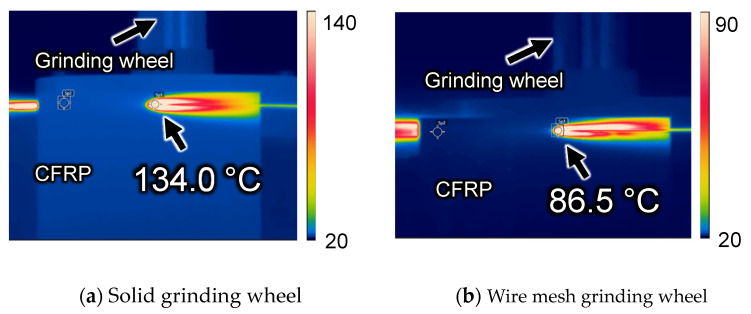
Maximum temperature measured by thermocamera.

Figure 18 shows the maximum temperature measured. The machining temperature of the solid wheel was 134 °C, whereas that of the wire mesh wheel was 86.5 °C, a difference of about 50 °C. This difference is attributable to two points. The first is the presence or absence of clogging. In the case of the solid grinding wheel, the machining temperature increased due to clogging, whereas the wire mesh grinding wheel maintained its sharpness and suppressed the increase in machining temperature. The second point is thought to be that the wire mesh grinding wheel has peripheral runout, which causes intermittent machining, resulting in a high temperature at one point on the wheel but a lower average temperature over the entire circumference of the wheel.

**Figure 19:**
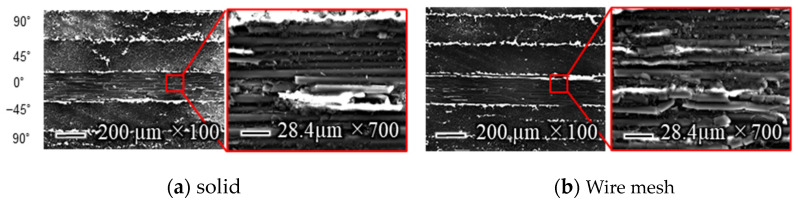
Workpieces observed by SEM.

Figure 19 shows the state of fibers and resin dissolution on the machined surface during high-speed cutting, as observed by scanning electron microscopy. When comparing the workpiece machined with a solid grinding wheel to the one machined with a wire mesh grinding wheel under a magnification of 100×, white areas can be observed on the workpiece machined with the solid grinding wheel. This can be attributed to the occurrence of charge-up due to the accumulation of electric charge on the nonconductive resin part of the workpiece. It is estimated that the resin underwent localized welding due to the temperature rise during processing. The glass transition temperature of the epoxy resin contained in the CFRP used in this experiment is approximately 180 °C. Because the material strength decreases above this temperature, it is important to control the temperature increase when processing CFRP. Although the thermographic camera measures the temperature of the grinding wheel surface, which is lower than the machining point, the actual temperature at the machining point is estimated to have been above 180 °C for the solid grinding wheel but below 180 °C for the wire mesh grinding wheel.

A comparison of the observation of the 0° fiber direction at 700× magnification shows that the workpiece machined with the wire mesh wheel had finer-cut carbon fibers than that machined with the solid wheel. This is presumably due to the low dimensional accuracy of the former, which prevents the abrasive grains that do the work from acting evenly on the surface to be machined. Grinding is a machining method in which a tool is given a certain amount of depth of cut, and the motion trajectory of the tool is transferred to the workpiece, so the accuracy of the tool’s depth of cut is directly reflected in the machining accuracy. In the case of a cutoff wheel, the runout in the z direction shown in Figure 1 affects the machining accuracy. During the measurement of runout in the z-direction, the solid grinding wheel exhibited a runout of less than 0.1 mm, while the wire mesh wheel had a runout of 0.8 mm. This indicates that the height of the abrasive grains on the work surface in the *z*-axis direction is not uniform, which means that some abrasive grains are cutting less or more than the desired depth of cut. This results in a nonuniform machined surface, as shown in Figure 19.

#### 4.1.4. Observation of Machined Surface and Surface Roughness

**Figure 20:**
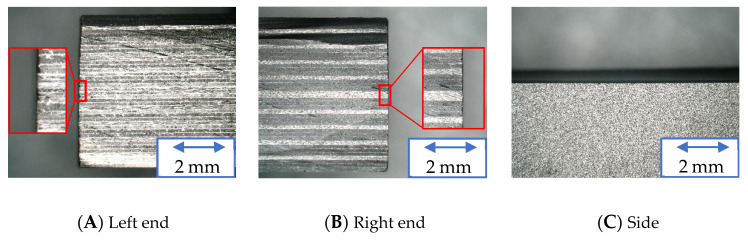
Machined surface (Resinoid tool).

**Figure 21:**
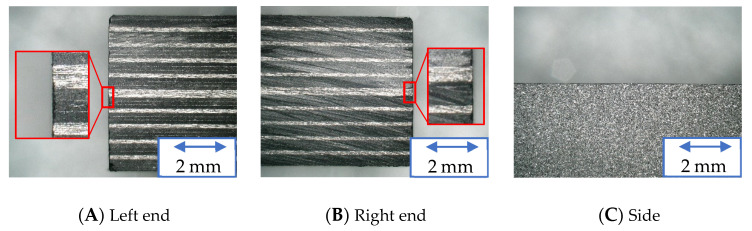
Machined surface (Solid tool).

**Figure 22:**
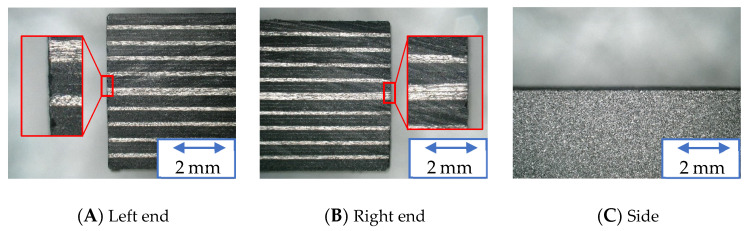
Machined surface (Wire mesh tool).

Figure 20, Figure 21 and Figure 22 show the machined surfaces of the workpieces under experimental condition A for the resinoid, solid, and wire mesh grinding wheels, respectively. Photographs of the left end, right end, and side of the cut workpiece are shown. The grinding wheel was fed from the left to the right of the workpiece. Under the conditions of this experiment, although slight burrs were observed at both ends, there were no noticeable delaminations or uncut fibers. There were no burrs on the sides.

**Figure 23:**
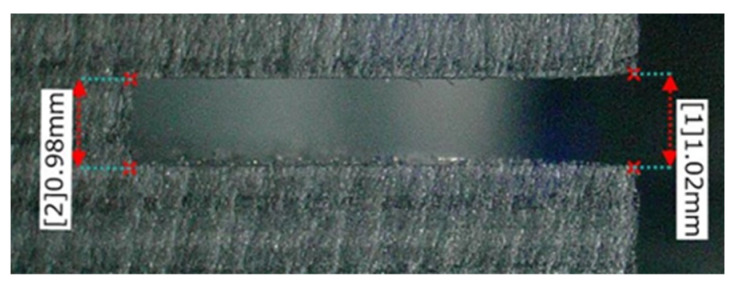
Machined groove (Resinoid tool).

**Figure 24:**
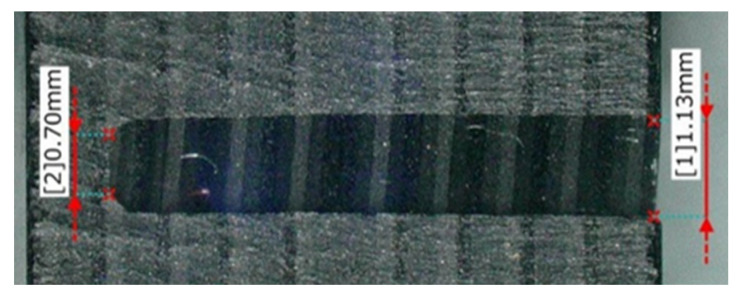
Machined groove (Solid tool).

**Figure 25:**
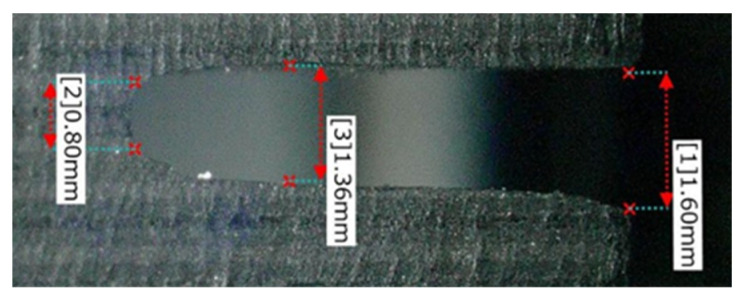
Machined groove (Wire mesh tool).

Figure 23, Figure 24 and Figure 25 show the grooves machined by the resinoid, solid, and wire mesh grinding wheels, respectively, under experimental condition A. The thickness of the grinding wheel was uniformly 1 mm. Groove widths were 1.02 mm when machined with a resinoid wheel, 1.13 mm with a solid wheel, and 1.6 mm with a wire mesh wheel. The groove width from the wire mesh wheel was 1.6 times larger than the thickness of the wheel. This is thought to be due to the runout in the z direction shown in Figure 1. As mentioned above, the wire mesh grinding wheel exhibited a runout of 0.8 mm, indicating that some abrasive grains were cutting beyond the desired depth of cut. As the feed rate increased, resulting in deterioration of the groove shape accuracy and surface roughness in general. However, straight cutting and grooving were possible even when the feed rate was increased up to about 2000 mm/min. On the other hand, with a tool with a 20 mm protrusion for deeper grooving, the wheel was deformed, and straight grooving was difficult even when the feed rate was 1000 mm/min. It also shows the importance of the stiffness of the wire mesh.

Table 3 shows the surface roughness values of the workpieces. While the arithmetic mean roughness of the workpiece machined with the solid grinding wheel was 1.37 µm, the wire mesh grinding wheel had a larger value, 2.76 µm. As mentioned above, this is thought to be due to the unevenness of the machined surface caused by the low dimensional accuracy of the wire mesh wheel. However, the required *Ra* ≤ 3.2 μm for specific aircraft parts is met. These results indicate that CFRP machined with a wire mesh wheel can be used to fabricate aircraft parts because no burr or delamination was observed, and the surface roughness met the standard value. However, attention should be paid to dimensional accuracy when machining grooves.

### 4.2. Tool Life Test with Continuous Grooving

**Figure 26:**
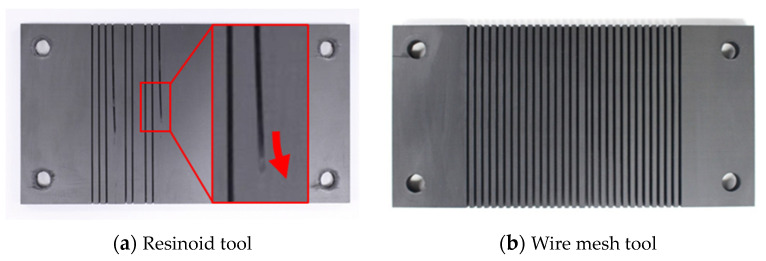
Workpieces after grooving.

Figure 26 shows the workpieces after machining. The solid and wire mesh grinding wheels completed 60 grooving operations on a 100 mm CFRP plate (machining distance: 6000 mm) without any problems, but the resinoid grinding wheel broke after the fourth cut (machining distance: 400 mm). Looking at the workpiece after machining, we found that the groove was curved at an angle when the resinoid grinding wheel broke. Under the axial load, the resinoid grinding wheel experienced slight elastic deformation. However, due to its limited ability to withstand the applied load, the wheel eventually fractured in a brittle manner.

Table 4 shows the outer diameter of each wheel before and after machining. For the resinoid grinding wheel, the outside diameter was measured after two cuts (machining distance: 200 mm), and for the solid and wire mesh grinding wheels, the outside diameter was measured after 60 cuts. The resinoid grinding wheel showed about 0.05 mm of wear after two cuts, while the solid and wire mesh grinding wheels showed almost no wear (less than 0.02 mm), even after 60 cuts. Because no significant difference was observed in the wear of those two grinding wheels, we compared their performance in terms of grain shedding and crushing.

**Figure 27:**
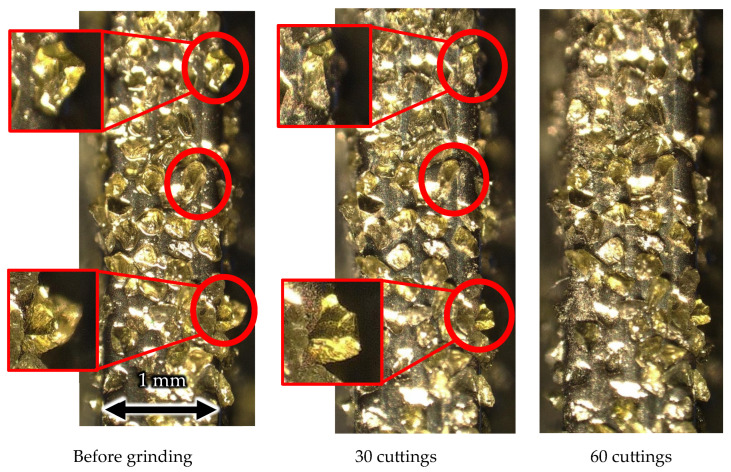
Surface of solid grinding wheel.

**Figure 28:**
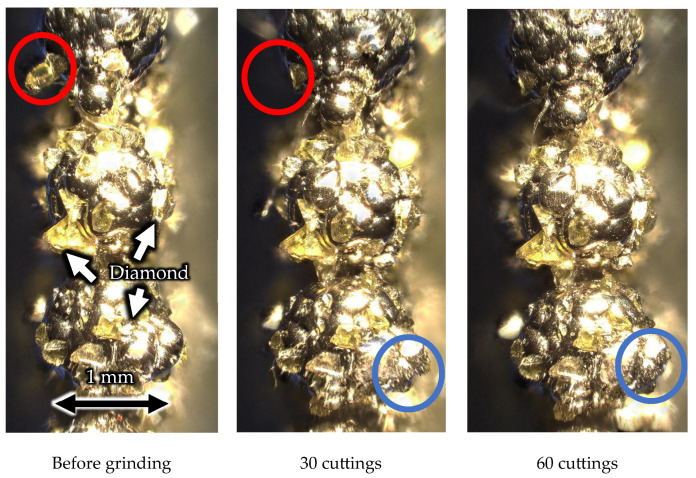
Surface of wire mesh grinding wheel.

Figure 27 and Figure 28 show the outer circumference areas involved in machining with the solid and wire mesh grinding wheels, respectively. The arrows indicate abrasive grains, and the other places are Ni-plated surfaces. From left to right, the figures show the state of the wire mesh grinding wheel prior to machining, after 30 cuts (machining distance: 3000 mm), and after 60 cuts (machining distance: 6000 mm), respectively. In the solid grinding wheel, the abrasive grains were crushed in the red circles during the first 30 cuts. After the next 30 cuts, we found no abrasive grain crushing or wear. On the other hand, for the wire mesh grinding wheel, the red circles indicate that the abrasive grains fell off during the first 30 cuts. Abrasive grains with a weak fixation force, which were not securely embedded within the plating layer, became dislodged and dropped out. This phenomenon is referred to here as “initial shedding”. The wire mesh grinding wheel showed only initial spilling, whereas the solid grinding wheel showed a lot of abrasive grains crushed even in the small area observed. We attribute this to the disparity in the modulus of elasticity between the two types of wheels.

As shown in Figure 8, the elastic modulus of the wire mesh wheel is 2.4 times smaller than that of the solid wheel and is easily deformed. The solid wheel has a high elastic modulus and is not easily deformed, so when a load is concentrated on the abrasive grains at the tip, the stress is high, and crushing occurs. On the other hand, the wire mesh wheel, characterized by a low modulus of elasticity, exhibits moderate deformation even under the application of impact processing forces. This deformation capability plays a crucial role in preventing excessive impact input to the diamond abrasive grains and is assumed to help prevent grain crushing.

In addition, a previous study [24] noted that wire mesh grinding wheels are self-sharpening. When abrasive grains at the tool tip become dislodged, it exposes a metal wire that is more prone to wear compared to the diamond grains. Once exposed, the wire is susceptible to immediate abrasion and quickly gets shaved off. As a result, the abrasive grains that exist on the sides of the grinding wheel appear at the tip, enabling machining by abrasive grains at all times. Although this phenomenon was not confirmed under the experimental conditions due to low wear, it is presumed that the low modulus of elasticity prevents the abrasive grains from fracturing; in addition, the self-sharpening prolongs tool life.

## 5. Conclusions

In this study, we proposed a tool with diamond abrasive grains electrodeposited on a disc-shaped wire mesh. The obtained results regarding the machining characteristics and tool life when performing deep grooving and cutoff of CFRP using this tool were as follows.

1. Because chips are discharged through the spaces in the mesh, clogging is avoided, and thus the machining temperature of the wire mesh wheel is lower than that of the resinoid or solid wheel.
2. CFRP machined with a wire mesh wheel can be used to fabricate aircraft parts because no burr or delamination was observed, and the surface roughness met the standard value, although attention should be paid to dimensional accuracy when machining grooves.
3. The wire mesh grinding wheel showed almost no wear after 6000 mm of machining. It has a low modulus of elasticity, so it deforms moderately even when an impact processing force is applied. This avoids impact input to the diamond abrasive grains and is assumed to avoid grain crushing.
4. It is estimated that tool life can be prolonged due to the absence of clogging, avoidance of abrasive grain crushing, and self-sharpening.

The greatest feature of the proposed tool is its ability to machine grooves with large aspect ratios over a long life. This paper describes grooving to a depth of 7 mm, but we have confirmed that grooving to a depth of 15 mm is also possible with a larger wheel. Current issues include the effects of vibration due to the low stiffness of the tool, which increases grinding resistance, and the low axial dimensional accuracy of the grinding wheel, which results in wide grooves. In the future, we plan to develop a highly rigid and precise tool by using a fabrication method that compresses several layers of wire mesh.

## Figures and Tables

**Table 1:** Mechanical properties of CFRP.

**Carbon Fiber**		Toho Tenax QU 135-197A
**Resin**		Epoxy resin #135
**Fabric Weight**	g/m^2^	190
**Curing Temperature**	°C	180
**Thickness of Prepreg**	mm	0.187
**Number of Layers**		40
**Thickness of Workpiece**	mm	7.6
**Length of Workpiece**	mm	100

**Table 2:** Machining conditions A and B.

**Length of Workpiece**	mm	1000	
**Grinding Speed *V***	m/min	1884	
**Feed Rate *f***	mm/min	A:100	B:1000
**Grinding Direction**		Down cut	
**Fluid Supplying Volume**	L/min	A:8.0	B:Dry

**Table 3:** Surface roughness.

	*Ra* (µm)	*Rz* (µm)
Resinoid	0.502	3.18
Solid	1.37	7.25
Wire mesh	2.76	11.5

**Table 4:** Diameter of each grinding wheel (mm).

	Resinoid Distance: 200 mm	Solid Distance: 6000 mm	Wire Mesh Distance: 6000 mm
Before cutting	150.85	150.32	151.34
After cutting	150.80	150.30	151.33
Wear of grinding wheel	−0.05	−0.02	−0.01

## Data Availability

Not applicable.
